# Supplementary material for: Development and validation of a multiplex immunoassay for the simultaneous quantification of type-specific IgG antibodies to E6/E7 oncoproteins of HPV16 and HPV18
Source: PLoS One. 2020 Mar 26;15(3):e0229672. doi: 10.1371/journal.pone.0229672 (PMC7098588; doi:10.1371/journal.pone.0229672)
Supplement: S1 Fig — UniProt derived and MBP-tagged sequences of synthesized HPV16/18 E6 and E7 proteins. (DOCX) [file pone.0229672.s001.docx]

**S1 Fig**

**MBP-HPV16 E6**

MKIEEGKLVIWINGDKGYNGLAEVGKKFEKDTGIKVTVEHPDKLEEKFPQVAATGDGPDIIFWAHDRFGGYAQSGLLAEITPDKAFQDKLYPFTWDAVRYNGKLIAYPIAVEALSLIYNKDLLPNPPKTWEEIPALDKELKAKGKSALMFNLQEPYFTWPLIAADGGYAFKYENGKYDIKDVGVDNAGAKAGLTFLVDLIKNKHMNADTDYSIAEAAFNKGETAMTINGPWAWSNIDTSKVNYGVTVLPTFKGQPSKPFVGVLSAGINAASPNKELAKEFLENYLLTDEGLEAVNKDKPLGAVALKSYEEELVKDPRIAATMENAQKGEIMPNIPQMSAFWYAVRTAVINAASGRQTVDEALKDAQTNSSSNNNNNNNNNNLGIEGRISHMGGSENLYFQGAGAPHQKRTAMFQDPQERPRKLPQLCTELQTTIHDIILECVYCKQQLLRREVYDFAFRDLCIVYRDGNPYAVCDKCLKFYSKISEYRHYSYSLYGTTLEQQYNKPLSDLLIRCINCQKPLSPEEKQRHLDKKQRFHNIRGRWTGRCMSCSRSSRTRRETQLHHHHHH

**MBP-HPV16 E7**

MKIEEGKLVIWINGDKGYNGLAEVGKKFEKDTGIKVTVEHPDKLEEKFPQVAATGDGPDIIFWAHDRFGGYAQSGLLAEITPDKAFQDKLYPFTWDAVRYNGKLIAYPIAVEALSLIYNKDLLPNPPKTWEEIPALDKELKAKGKSALMFNLQEPYFTWPLIAADGGYAFKYENGKYDIKDVGVDNAGAKAGLTFLVDLIKNKHMNADTDYSIAEAAFNKGETAMTINGPWAWSNIDTSKVNYGVTVLPTFKGQPSKPFVGVLSAGINAASPNKELAKEFLENYLLTDEGLEAVNKDKPLGAVALKSYEEELVKDPRIAATMENAQKGEIMPNIPQMSAFWYAVRTAVINAASGRQTVDEALKDAQTNSSSNNNNNNNNNNLGIEGRISHMGGSENLYFQGAGAPHGDTPTLHEYMLDLQPETTDLYCYEQFNDSSEEEDEIDGPAGQAEPDRAHYNIVTFCCKCDSTLRLCVQSTHVDIRTLEDLLMGTLGIVCPICSQKPHHHHHH

**MBP-HPV18 E6**

MKIEEGKLVIWINGDKGYNGLAEVGKKFEKDTGIKVTVEHPDKLEEKFPQVAATGDGPDIIFWAHDRFGGYAQSGLLAEITPDKAFQDKLYPFTWDAVRYNGKLIAYPIAVEALSLIYNKDLLPNPPKTWEEIPALDKELKAKGKSALMFNLQEPYFTWPLIAADGGYAFKYENGKYDIKDVGVDNAGAKAGLTFLVDLIKNKHMNADTDYSIAEAAFNKGETAMTINGPWAWSNIDTSKVNYGVTVLPTFKGQPSKPFVGVLSAGINAASPNKELAKEFLENYLLTDEGLEAVNKDKPLGAVALKSYEEELVKDPRIAATMENAQKGEIMPNIPQMSAFWYAVRTAVINAASGRQTVDEALKDAQTNSSSNNNNNNNNNNLGIEGRISHMGGSENLYFQGAGAPARFEDPTRRPYKVPDLCTELNTSLQDIEITCVYCKTVLELTEVFEFAFKDLFVVYRDSIPHAACHKCIDFYSRIRELRHYSDSVYGDTLEKLTNTGLYNLLIRCLRCQKPLNPAEKLRHLNEKRRFHNIAGHYRGQCHSCCNRARQERLQRRRETQVHHHHHH

**MBP-HPV18 E7**

MKIEEGKLVIWINGDKGYNGLAEVGKKFEKDTGIKVTVEHPDKLEEKFPQVAATGDGPDIIFWAHDRFGGYAQSGLLAEITPDKAFQDKLYPFTWDAVRYNGKLIAYPIAVEALSLIYNKDLLPNPPKTWEEIPALDKELKAKGKSALMFNLQEPYFTWPLIAADGGYAFKYENGKYDIKDVGVDNAGAKAGLTFLVDLIKNKHMNADTDYSIAEAAFNKGETAMTINGPWAWSNIDTSKVNYGVTVLPTFKGQPSKPFVGVLSAGINAASPNKELAKEFLENYLLTDEGLEAVNKDKPLGAVALKSYEEELVKDPRIAATMENAQKGEIMPNIPQMSAFWYAVRTAVINAASGRQTVDEALKDAQTNSSSNNNNNNNNNNLGIEGRISHMGGSENLYFQGAGAPHGPKATLQDIVLHLEPQNEIPVDLLCHEQLSDSKEENDEIDGVNHQHLPARRAEPQRHTMLCMCCKCEARIELVVESSADDLRAFQQLFLNTLSFVCPWCASQQHHHHHH

MBP

Linker, protease sites (FXa, TEV)

HPV E6/E7 + His tag
