# Supplementary material for: Development and validation of a multiplex immunoassay for the simultaneous quantification of type-specific IgG antibodies to E6/E7 oncoproteins of HPV16 and HPV18
Source: PLoS One. 2020 Mar 26;15(3):e0229672. doi: 10.1371/journal.pone.0229672 (PMC7098588; doi:10.1371/journal.pone.0229672)
Supplement: S1 Raw images — (PDF) [file pone.0229672.s004.pdf]

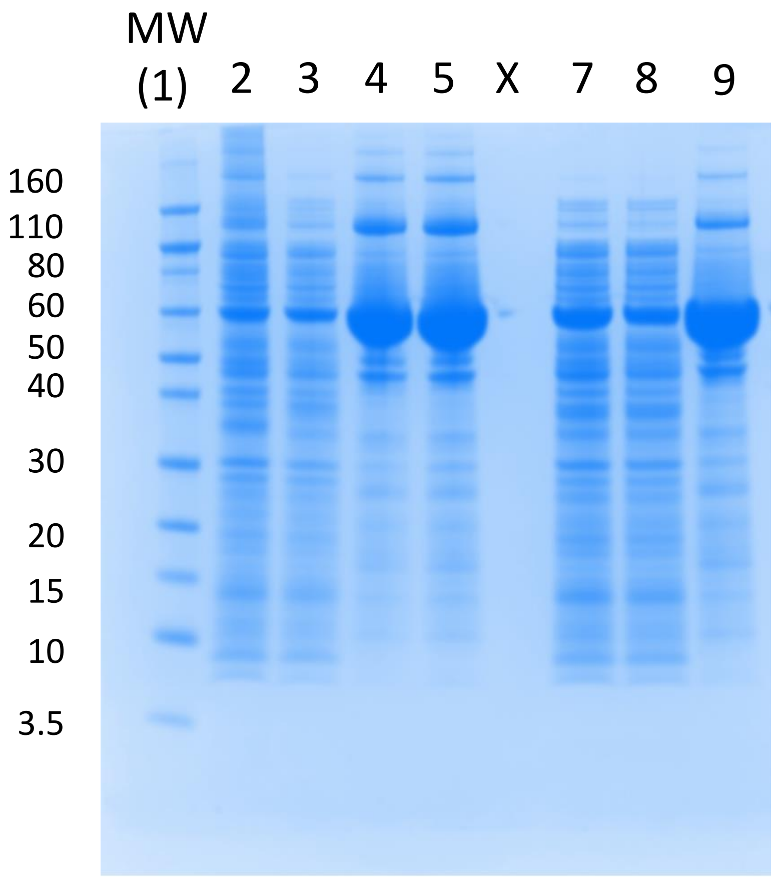

Figure 1a gel. Lane 1 designated the molecular weight marker, lane 2 is protein loaded on the column, lane 3 is the flow-through from the column, lanes 4 and 5 are independent collections of the peak of the primary elution, lane 6 is empty, lanes 7-9 is raw load protein (7), column flow-through (8), and peak of the primary elution (9) subjected to DTT reduction. Image captured using ChemiDoc XRS (BioRad; Hercules, CA, with exposure time <1 sec.

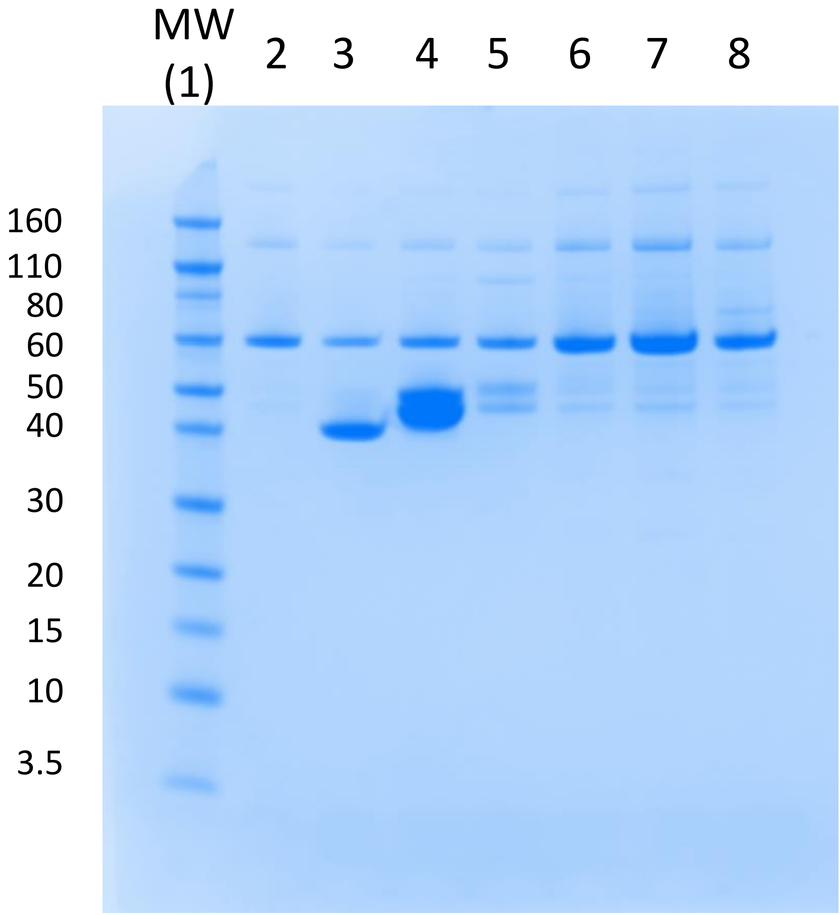

Figure 1B gel. Lane 1 designated the molecular weight marker, lane 2 is protein loaded on the column, lane 3 is the flow-through from the column, lanes 4 and 5 are independent collections of the peak of the primary elution, lanes 6-8 is raw load protein (6), column flow-through (7), and peak of the primary elution (8) subjected to DTT reduction. Image captured using ChemiDoc XRS (BioRad; Hercules, CA, with exposure time <1 sec.
