# Supplementary material for: Development and validation of a multiplex immunoassay for the simultaneous quantification of type-specific IgG antibodies to E6/E7 oncoproteins of HPV16 and HPV18
Source: PLoS One. 2020 Mar 26;15(3):e0229672. doi: 10.1371/journal.pone.0229672 (PMC7098588; doi:10.1371/journal.pone.0229672)
Supplement: Attachment — Submitted filename: Response to Reviewers.docx [file pone.0229672.s005.docx]

**Rebuttal Letter**

We appreciate the thorough review from Reviewers 1 and 2 and their comments regarding the quality of the data and to more clearly state our abstract and conclusions in the discussion. Below, we capture the comments from each reviewer and respond with the following edits that can be found in the marked-up text document.

**Editor Comments:**

Style requirements for file naming – addressed

Clarify whether human and monkey specimens were obtained from third parties commercial or not. Human samples were obtained from BioIVT (Hicksville NY) or Proteogenex (Inglewood, CA) with informed consent. This has been updated in the cover letter. The monkey serum samples were obtained as a gift from Jean Boyer and the animal protocol was approved with IACUC Board approval at Inovio Pharmaceuticals in San Diego, CA.

Funding statements and competing interests have been updated for all authors in the cover letter.

**Reviewer 1**:

1. Several lines of evidence suggest presence of cross-reactivity in the assay. Given this, not only does it appear that “… we cannot rule out the cross-reactive nature of HPV18 and HPV18 E6 antibodies.” But, one might “rule in” the likely presence of such an effect. The abstract and discussion should be more upfront about this fact.

We address the cross-reactivity effect upfront in the abstract more clearly (line 32 and 33) by stating that HPV18E6 is as good as neutralizing HPV16 E6 in 5 of 6 samples tested in the cross-reactivity experiment. Before it merely stated that cross-reactivity existed and was observed. Lines 288-290 now address this observation in the results. However, for the discussion, this is addressed in lines 480-485. We feel that the development of the assay and the metrics for qualification against a reference serum is most important, and that these observations could be found in sample testing to identify the reference serum. We did include a brief summary (lines 429-430) regarding the finding of the cross-reactivity up front in the discussion.

2. No mention is made in the abstract and discussion regarding the high detection of antibodies in pediatric (presumably unexposed samples), which is suggestive of some non-specificity of the assay (Figure 4). In fact, average concentrations, between 100-1,000 were noted for all antigens except for HPV18 E7. This high detection of signal among individuals expected to be unexposed, together with the increased dynamic range of the assay, suggest that the authors’ attempt to increase dynamic range (decrease the LLOQ) might have led to reduced specificity of the assay.

We acknowledge the levels of basal activity in what we would presume would be an unexposed group to HPV. We have addressed this by updating the methods to include the age of the diagnostic retains of pediatric samples (1-5 day old infants; line 98). Having this information is important as maternal antibodies would be detected at this age and they may be at low concentrations. We have updated the text in the results to reflect this finding as well (lines 312-314) and included a reference from a CVI paper Matys et al from 2012 (PMID 22518014). This was also addressed in the discussion in lines 444-446.

3. The final sentence of the abstract is a bit of an overstatement since comparison with ELISA was performed for only one of the four antigens included in the assay.

We have removed the language regarding standard ELISA methods (lines 35-39) to show what we have really developed: an assay that is high throughput, multiplexed, a good dynamic range, and sample input.

4. The fourth paragraph of the discussion seems speculative and is not based on data reported in the results. This reviewer suggests dropping that paragraph, which makes statements about the advantage of the newly developed assay vis-à-vis other existing platforms (including multiplex platforms) without providing any data to support such assertions.

We feel that this section is necessary to describe the tag and recovery portion of the protein. Many other groups have tried other tags to HPV such as His, myc, etc and their results have not been as reproducible. We acknowledge this section is somewhat speculative and would require additional chemistry analysis but would be beyond the scope of the current paper.

**Reviewer #2**: The authors developed a specific, sensitive, reproducible multiplex serology assay for the simultaneous quantitation of IgG antibodies to HPV16/18 E6 and E7. The data are convincing. However, although the assay seems to be a good alternative to standard ELISA, the authors should better explain how this technology may be used as an alternative to the Luminex technology. Bead-based assays are widely used and demonstrate high performance in large-scale epidemiology studies (PMID: 16099939). Why the authors didn’t compare the performance of their assay with a bead-based assay (or serum previously characterized using a bead-based assay)? What about the cost for each sample? Also, the authors should explain better this technology on its way to detect specifically each of the antigens. It is not clear.

Point 1 – Why did the authors not compare the performance of their assay with a bead-based assay?

A previous study has evaluated a large cohort comparison of serum samples to HPV 6, 11, 16 and 18 L1 between MSD and Luminex (PMID: 25554636). We acknowledge there is a gap in our reagents toward their performance in a bead based assay. However, EDC crosslinking of proteins to beads may result in untoward effects of the labile HPV proteins. Because the MSD assay affords the protein to bind to the plate in its native confirmation post-synthesis, no chemical modifications are done to perturb the chemical structure. Text was added to the introduction (lines 69,70 and 75,76) to clarify the choice of MSD over Luminex and why we elected to not pursue Luminex.

Point 2 – What about the cost for each sample?

The cost of the sample was omitted from the manuscript because it does not contribute to the strength of the paper. However Luminex and MSD are comparable in cost. Roughly speaking MSD and Luminx would be around $200 per sample. Luminex would tend to be a little higher due to conjugation chemistry for proteins/antigens. 32 samples could be run per plate (in duplicate) with a standard curve in triplicate. For ELISA, sample cost would be significantly less, about $75/sample, however without the ability to multiplex, the cost per sample increases to $300+ and a much larger volume is needed. For precious samples, this could be a severe limitation.

Point 3 – The authors should better explain this technology on its way to detect specifically each of the antigens.

This is highlighted from point 1 in so far that the confirmation of the protein/antigen to the plate and its confirmation being conserved whereas with Luminex, conjugation chemistry may result in protein degradation.

Point 4 – Lines 434-435; the authors found similar antibody concentrations in HPV+ subjects to normal healthy donors which is surprising as only antibodies to L1 should be found. Please explain. The E6 antibodies should be rare in the general population.

We do agree that the antibody concentrations are similar, however this analysis needs to be expanded over a larger sample size to get true statistical differences and the scope of the manuscript is to report on the method development, and, secondarily, to identify who may be seropositive. We have adjusted the text to reflect the data shown in Fig 4. We did observe elevated median concentrations of E6 antibodies in patients with HPV+ infections (HPV16 and 18) of 1.5 – 2.2 fold (lines 317-320). We cannot rule out that the adult donor population may have an HPV infection not linked to cancer due to the source of our adult donor cohort which was unspecified for the number of sexual partners. It has been noted previously that women with 4+ sexual partners had seropositivity to HPV antibodies in >60% of serum evaluated (PMID: 11724843). We updated our conclusion (lines 447-453) to reflect the need to identify a truly negative population in additional assay development.
