# Supplementary material for: Development and validation of a multiplex immunoassay for the simultaneous quantification of type-specific IgG antibodies to E6/E7 oncoproteins of HPV16 and HPV18
Source: PLoS One. 2020 Mar 26;15(3):e0229672. doi: 10.1371/journal.pone.0229672 (PMC7098588; doi:10.1371/journal.pone.0229672)
Supplement: Attachment — Submitted filename: Response to Reviewers - Second Revision.docx [file pone.0229672.s006.docx]

**Rebuttal Letter for Second Revision**

From the second round of reviews following primary revision, all comments and concerns were addressed from Reviewer #2, however reviewer #1 has a concern regarding the interpretation of cross-reactivity and false positives. Specifically: “In addressing the higher than expected positivity observed among pediatric subjects, the authors are correct in stating that one explanation is the presence of maternal antibodies. However, they fail to explicitly state that an alternative explanation is lack of assay specificity (i.e., pediatric positives are false positives). This should be explicitly stated in the manuscript.” We accept this as a true limitation of the current assay and an epidemiological study to identify cutoff levels would serve this assay significantly.

We have now included a statement regarding this in the manuscript in the marked-up copy on lines 476-477. We do agree that the false positives could be from other sources due to polyclonal antibody affinity and avidity. A much larger HPV-positive and -negative cohort would aid us in identifying true serostatus levels and that is a limitation of the current assay as it is presented.
